# Supplementary material for: M(II)Al4 Type Layered Double Hydroxides—Preparation Using Mechanochemical Route, Structural Characterization and Catalytic Application
Source: Materials (Basel). 2021 Aug 27;14(17):4880. doi: 10.3390/ma14174880 (PMC8432663; doi:10.3390/ma14174880)
Supplement: Supplementary file 1 [file materials-14-04880-s001.zip › materials-1354801-supplementary.pdf]

materials

Supplementary Information

M(II)Al4 Type Layered Double Hydroxides—Preparation Using Mechanochemical Route, Structural Characterization and Catalytic Application

Márton Szabados [1,2], Adél Anna Ádám [2], Zsolt Kása [2], Kornélia Baán [3], Róbert Mucsi [3], András Sápi [3], Zoltán Kónya [3,4], Ákos Kukovecz [3] and Pál Sipos [2,5,*]

1 Department of Organic Chemistry, University of Szeged, Dóm tér 8, H-6720 Szeged, Hungary; szabados.marton@chem.u-szeged.hu
2 Material and Solution Structure Research Group, Institute of Chemistry, University of Szeged, Aradi Vértanúk tere 1, H-6720 Szeged, Hungary; adeladam@chem.u-szeged.hu (A.A.Á.); kasa.zsolt@chem.u-szeged.hu (Z.K.)
3 Department of Applied and Environmental Chemistry, University of Szeged, Rerrich B. tér 1, H-6720 Szeged, Hungary; kornelia.baan@chem.u-szeged.hu (K.B.); muule93@gmail.com (R.M.); sapia@chem.u-szeged.hu (A.S.); konya@chem.u-szeged.hu (Z.K.); kakos@chem.u-szeged.hu (Á.K.)
4 MTA-SZTE Reaction Kinetics and Surface Chemistry Research Group, Rerrich B tér 1, H-6720 Szeged, Hungary
5 Department of Inorganic and Analytical Chemistry, University of Szeged, Dóm tér 7, H-6720 Szeged, Hungary
* Correspondence: sipos@chem.u-szeged.hu

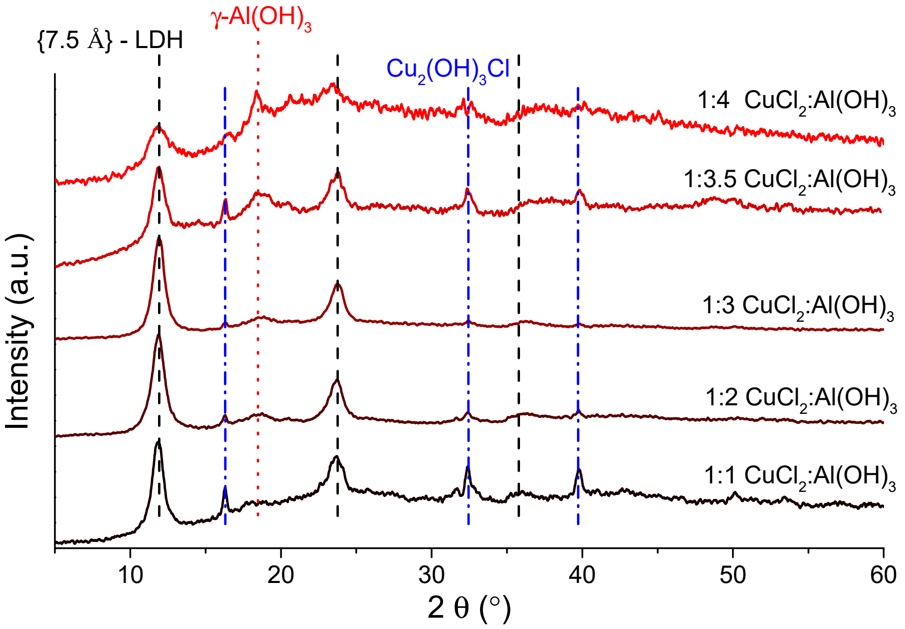

Figure S1. XRD patterns of the $CuAl_4$–Cl^-–LDH solids obtained using different initial Cu:Al molar ratios (reaction conditions: 96 h stirring, 90 °C, 6 h pre-milling). The basal spacing is shown in curly brackets.

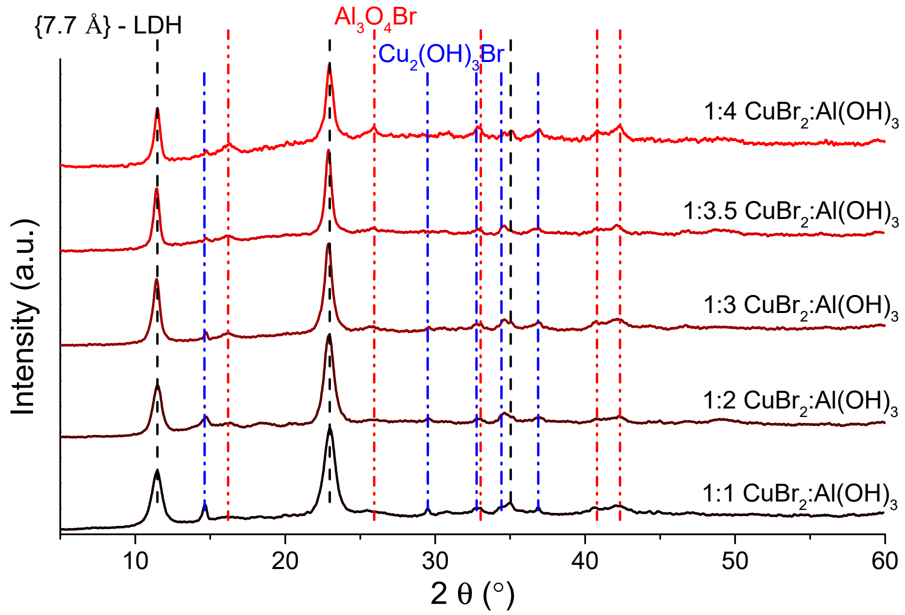

Figure S2. XRD patterns of the $CuAl_4$–Br^-–LDH solids obtained using different initial Cu:Al molar ratios (reaction conditions: 96 h stirring, 90 °C, 6 h pre-milling). The basal spacing is shown in curly brackets.

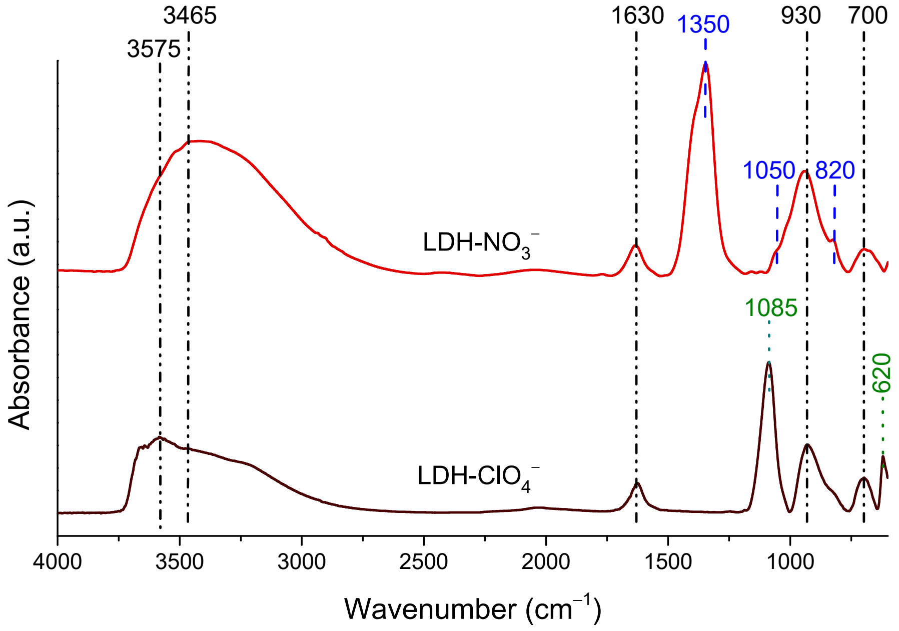

Figure S3. Infrared spectra of the $CuAl_4$–LDHs, prepared with nitrate and perchlorate interlayer anions.

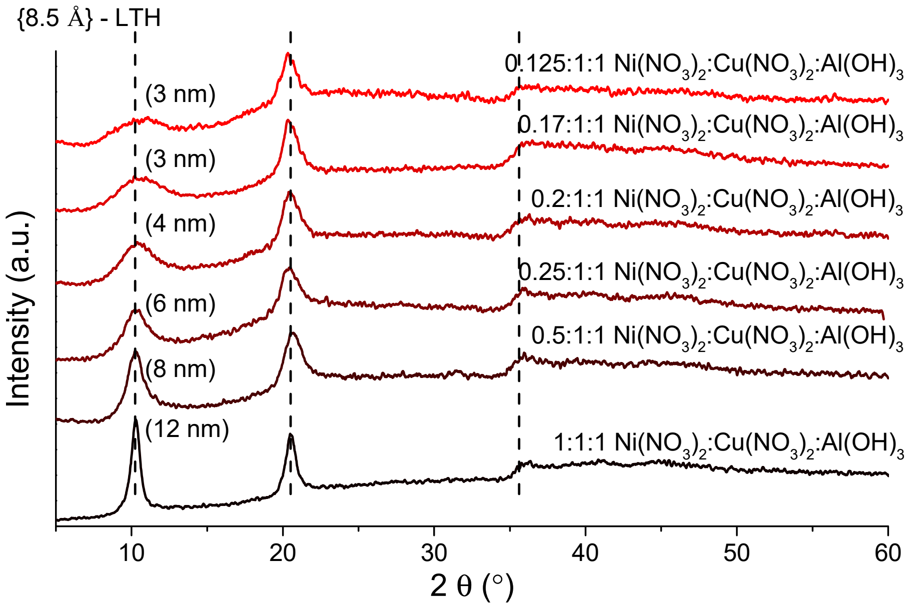

Figure S4. X-ray diffraction traces of the NiCuAl4–NO_3^-–LTH solids, with various initial Ni:Cu molar ratios (gradual decrease of the added Ni(II) amount, crystallite thicknesses shown in nm, reaction conditions: 96 h stirring period, 90 °C, 6 h pre-milling). The basal spacing is shown in curly brackets.

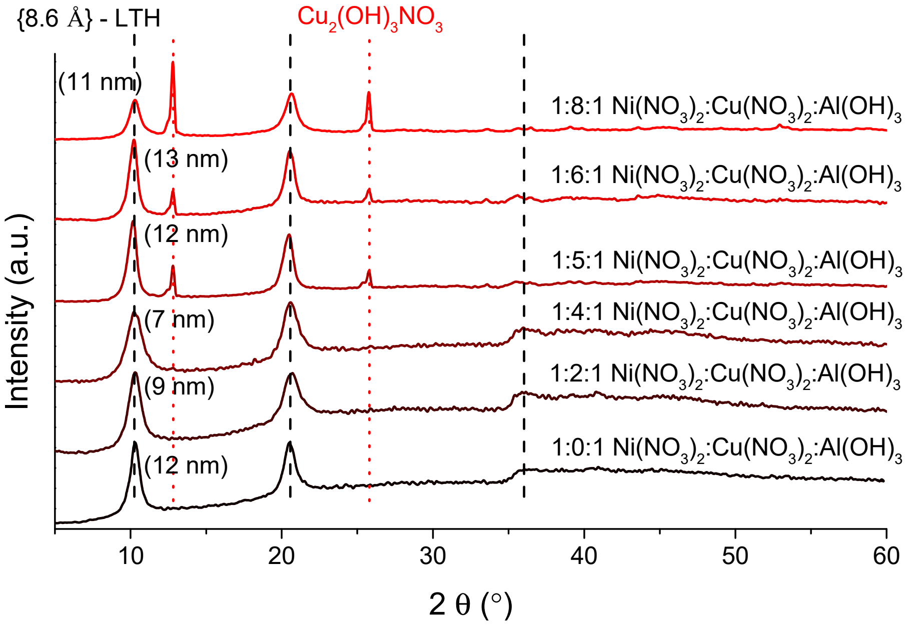

Figure S5. XRD patterns of the NiCuAl4–NO_3^-–LTH materials, with different initial Ni:Cu molar ratios (gradual increase of the added Cu(II) concentration, crystallite thicknesses denoted in nm, reaction conditions: 96 h stirring period, 90 °C, 6 h pre-milling). The basal spacing is shown in curly brackets.

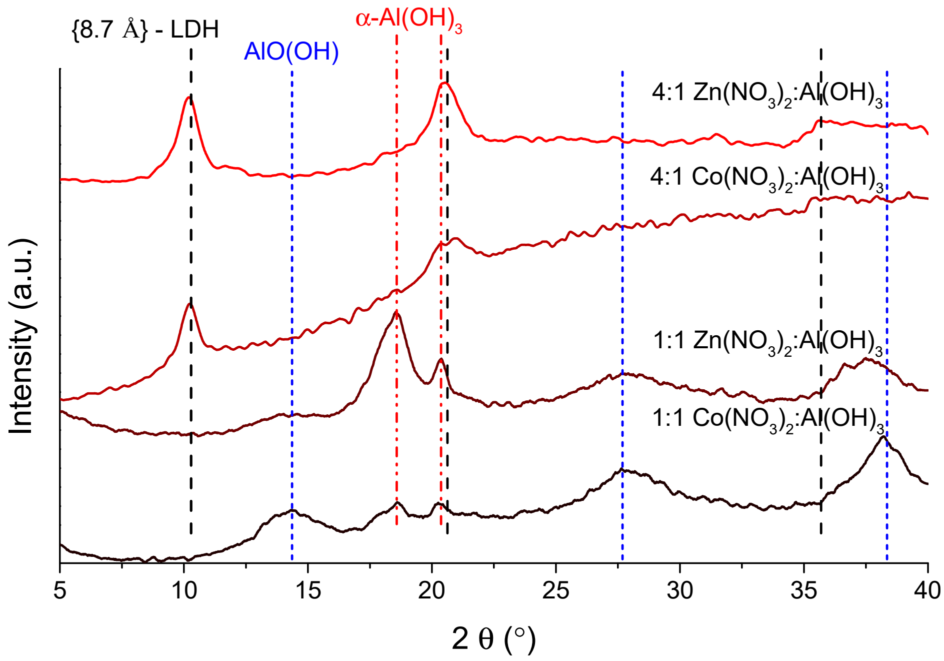

Figure S6. XRD curves of the $CoAl_4$– and $ZnAl_4$–NO_3–LDHs, with different initial Co/Zn:Al molar ratios (reaction conditions: 96 h stirring period, 90 °C, 6 h pre-milling). The basal spacing is shown in curly brackets.

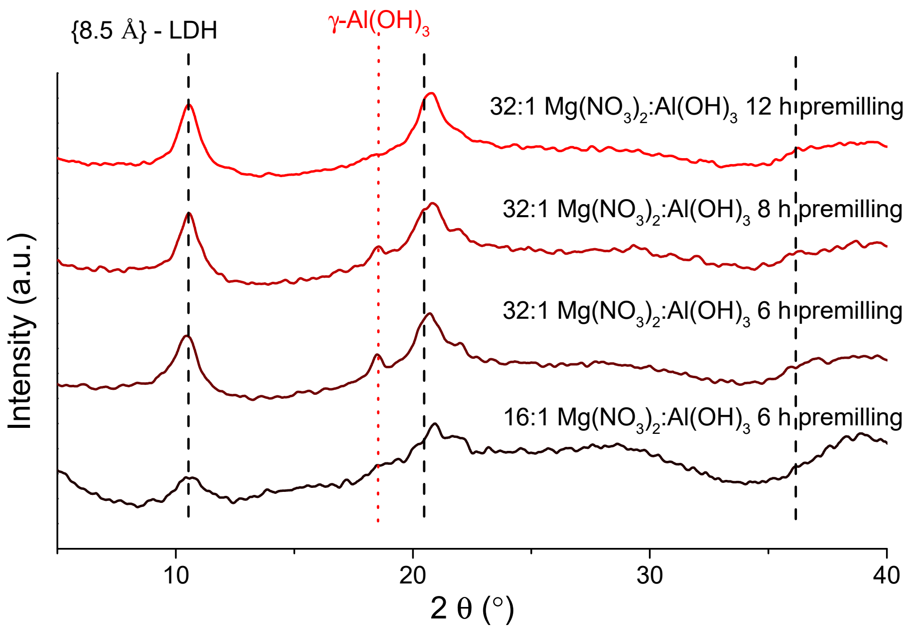

Figure S7. XRD patterns of the $MgAl_4$–NO_3^-–LDH solids, with various milling times and initial Mg:Al molar ratios (reaction conditions: 96 h stirring period, 90 °C). The basal spacing is shown in curly brackets.

Table S1. The molar ratios of the incorporated metal ions into the gibbsite structure; the initial and the measured values in the formed LTHs/LMHs are shown with perchlorate interlamellar anions.

Samples (LDH-ClO4)	Initial Molar Ratios[1]					Measured Molar Ratios				
	Mg	Ni	Co	Cu	Zn	Mg	Ni	Co	Cu	Zn
NiCu-Al	-	2	-	2	-	-	4.32	-	1	-
	-	1	-	2	-	-	2.51	-	1	-
	-	1	-	4	-	-	1.48	-	1	-
NiCo-Al	-	2	2	-	-	-	20.70	1	-	-
NiZn-Al	-	2	-	-	2	-	15.16	-	-	1
CoCu-Al	-	-	2	2	-	-	-	1	7.27	-
CuZn-Al	-	-	-	2	2	-	-	-	5.63	1
NiCoCu-Al	-	2	2	2	-	-	20.89	1	8.27	-
NiCoCuZn-Al	-	2	2	2	2	-	15.20	1	4.81	1.15
MgNiCoZnCu-Al	2	2	2	2	2	0.04	16.83	1	4.83	1.22

[1] The initial molar ratio of the aluminum was at 1 in every case.

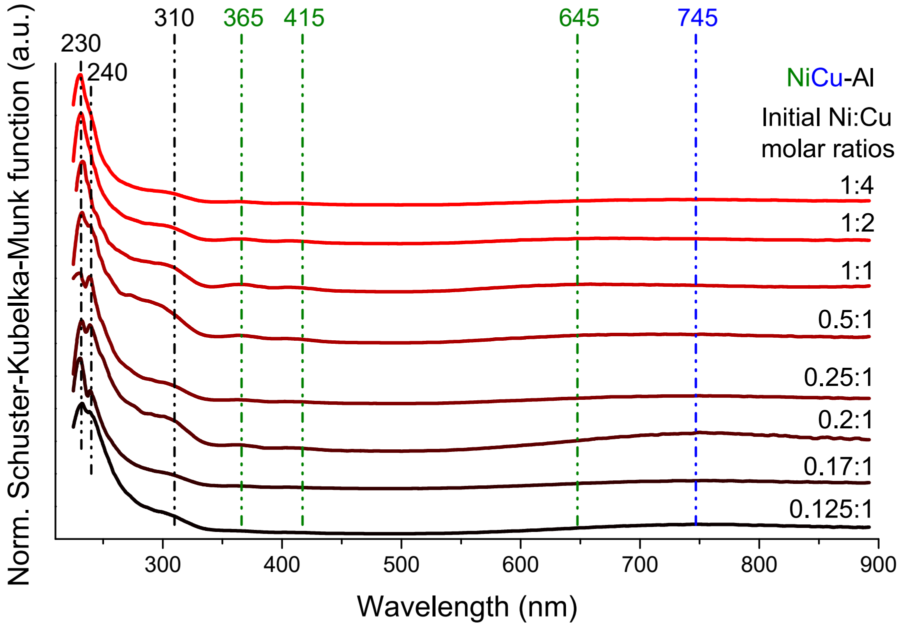

Figure S8. UV-Vis diffuse reflection spectra of the NiCuAl–LTHs, prepared with various initial Ni:Cu molar ratios.

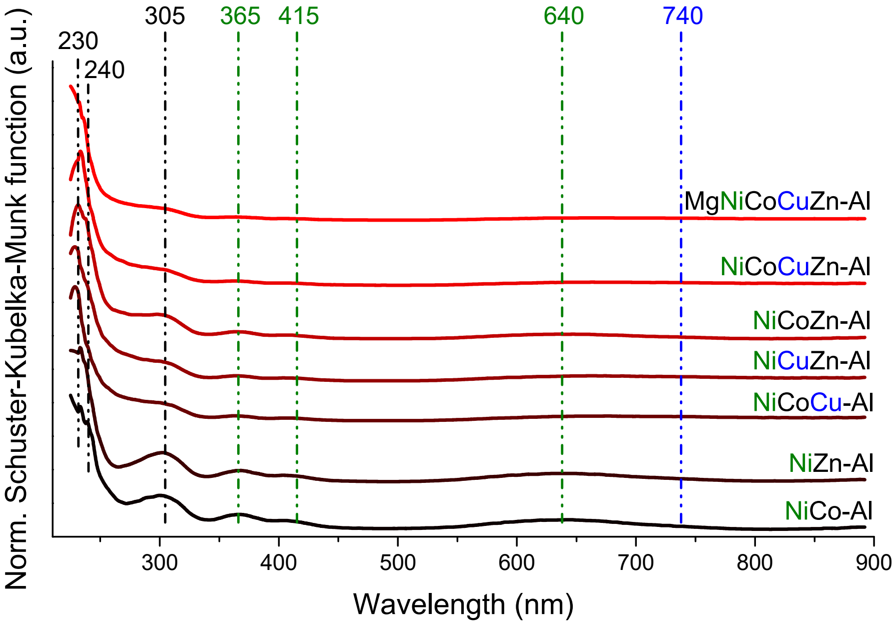

Figure S9. UV-Vis diffuse reflection spectra of nickel-containing LTHs/LMHs.

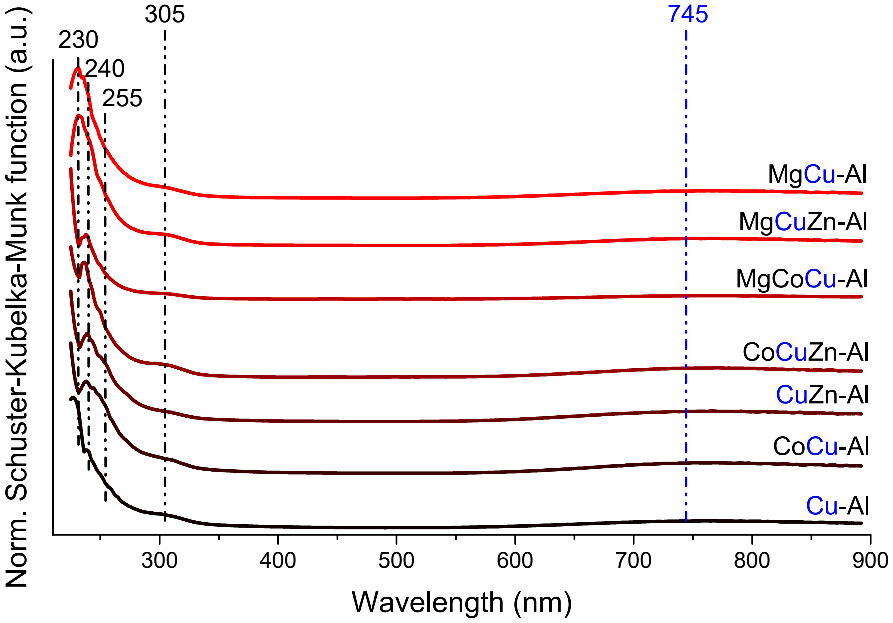

Figure S10. UV-Vis-DR spectra of copper-containing materials.

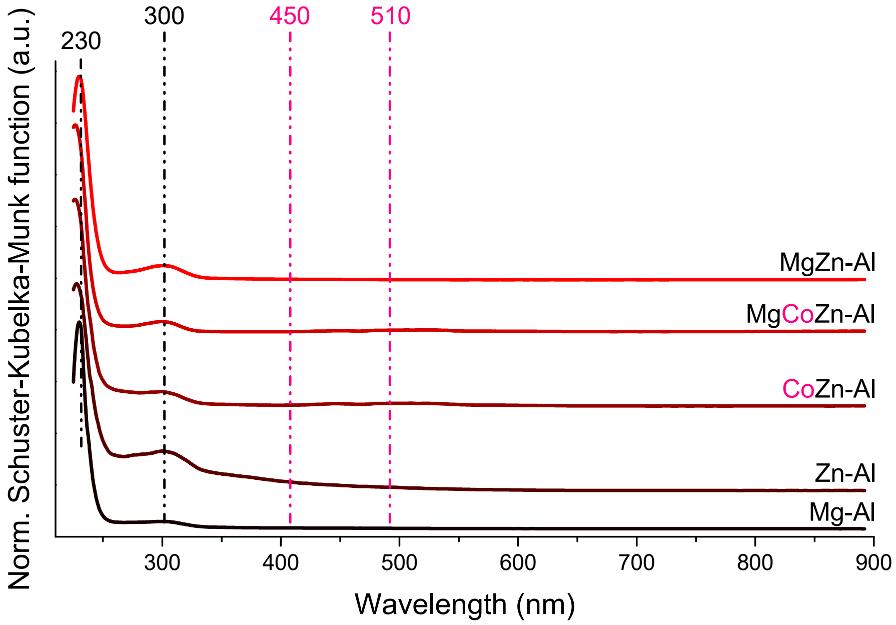

Figure S11. UV-Vis diffuse reflection spectra of zinc-containing solids and the $MgAl_4$–LDH.

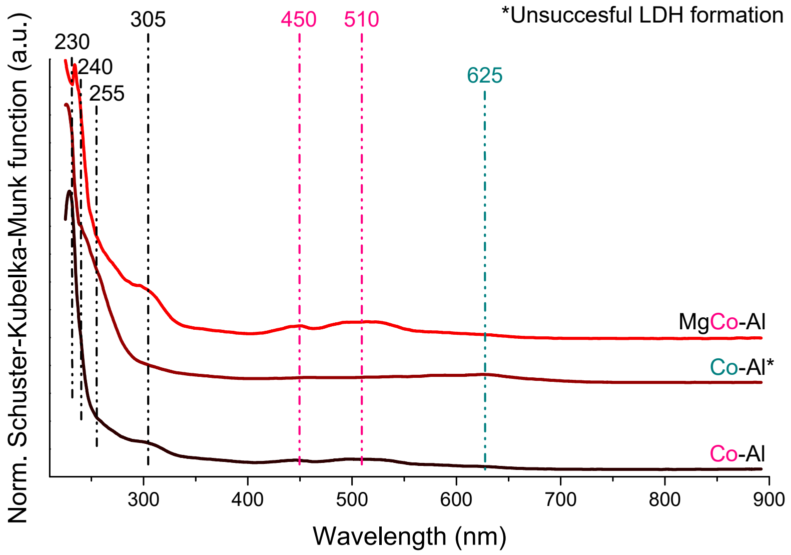

Figure S12. UV-Vis-DR spectra of cobalt-containing samples; for the failed CoAl–LDH synthesis (marked with an asterisk), the reaction conditions were the following: 1:1 initial Co:Al molar ratio, 96 h stirring period, 90 °C, 6 h pre-milling.

Table S2. Optical properties of the prepared LDHs.

Samples	Direct Band Gap (eV)	Indirect Band Gap (eV)
6 h milled $Al(OH)_3$	5.30	5.11
$NiAl_4$–LDH	5.07	4.71
$CuAl_4$–LDH	4.79	3.93
$CoAl_4$–LDH	5.02	4.67
$ZnAl_4$–LDH	4.97	4.53
$MgAl_4$–LDH	5.17	4.89

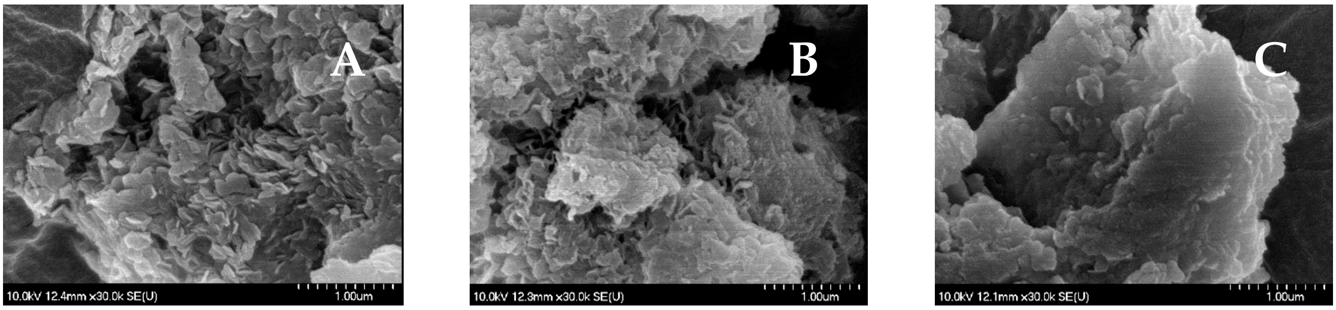

Figure S13. SEM images of the $ZnAl_4$– (A), $MgAl_4$– (B) and $CoAl_4$–LDH (C) prepared with nitrate interlamellar anions.

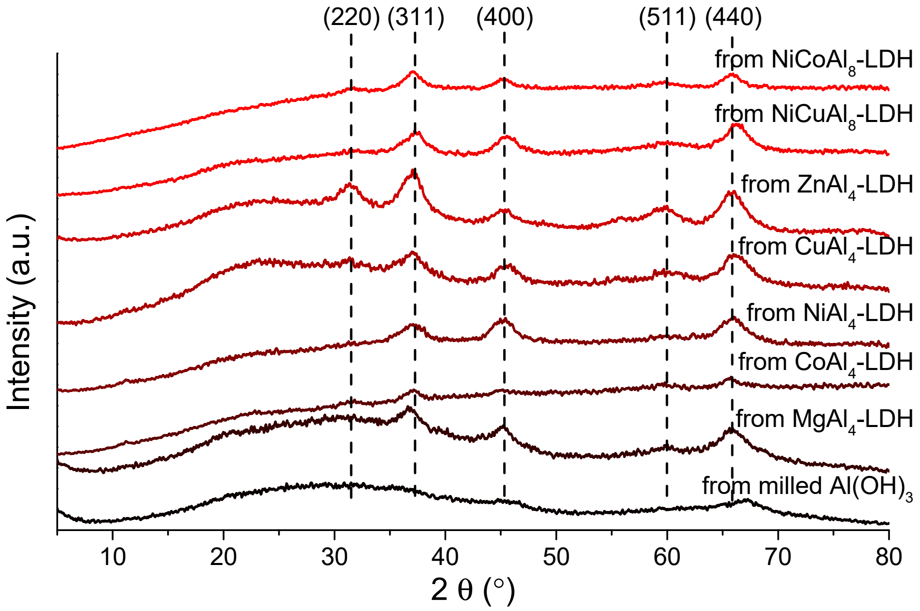

Figure S14. Powder X-ray diffraction patterns of the spent catalysts after long-term carbon monoxide oxidation at a 700 °C reaction temperature.

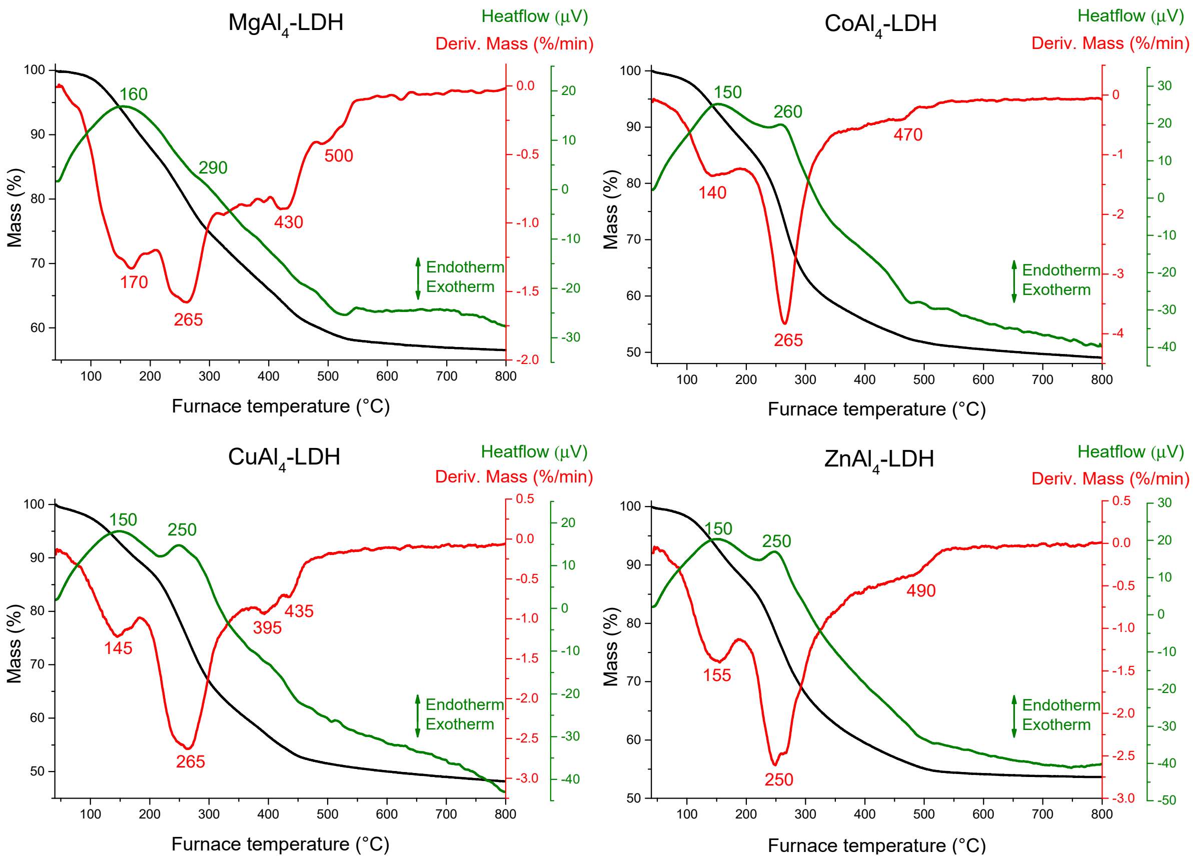

Figure S15. Thermogravimetric, derivative thermogravimetric and differential thermal analysis curves of the magnesium-, cobalt-, copper- and zinc-containing LDHs prepared with nitrate interlamellar anions.

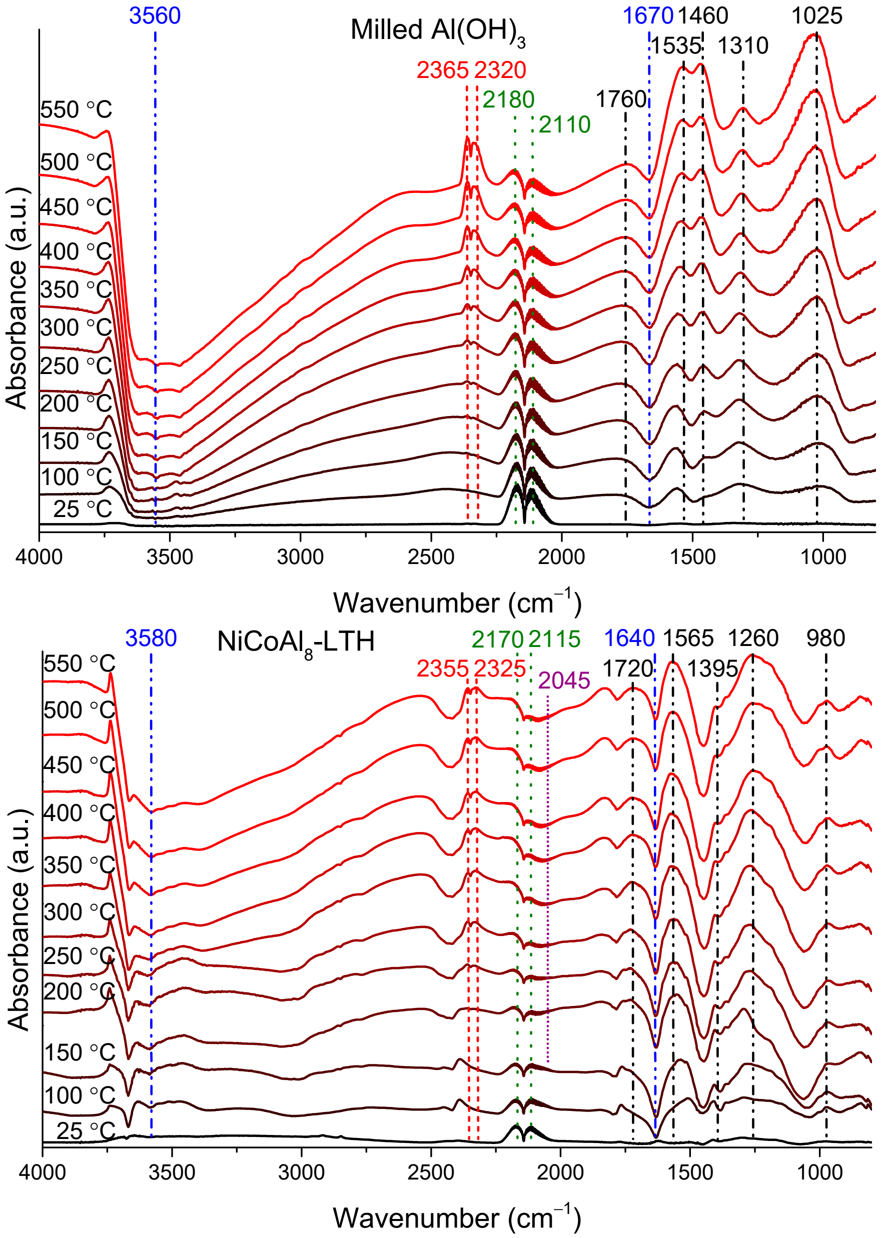

Figure S16. DRIFT spectra of the milled $Al(OH)_3$ and $NiCoAl_8$–LTH, heated up to 550 °C in the presence of a carbon monoxide–helium flow.
